# The role and importance of scientific training in medical school and other healthcare specialties

**DOI:** 10.31744/einstein_journal/2025ED1

**Published:** 2025-09-29

**Authors:** Luiz Vicente Rizzo, Edécio Cunha-Neto

**Affiliations:** 1 Hospital Israelita Albert Einstein São Paulo SP Brazil Hospital Israelita Albert Einstein, São Paulo, SP, Brazil.; 2 Universidade de São Paulo Faculdade de Medicina São Paulo SP Brazil Faculdade de Medicina, Universidade de São Paulo, São Paulo, SP, Brazil.

The issue of scientific training – scientific initiation (SI) in Brazil – for medical students is as old as evidence-based medicine. It goes far beyond training a new generation of physician-scientists needed for the continuous advancement of human health care. Advancements that throughout the 20^th^ century led to doubling the average life expectancy, by saving lives in childhood and prolonging life in adults and the elderly through prophylaxis and treatment of deadly diseases. Science training is crucial for medical students, as it equips them with fundamental skills for their careers. It fosters an inquisitive mindset that urges questions about the validity and appropriateness of any health intervention and enhances their ability to critically evaluate medical information. Scientific training helps them become better physicians by enhancing their critical thinking, problem-solving ability, and analytical skills. Furthermore, it prepares them for the constant need to stay up-to-date with advances in knowledge.^( 1 , 2 )^

Research training involves identifying gaps in medical knowledge compared to what is available in the scientific literature, formulating research questions, defining a working hypothesis and methodology, experimenting and analyzing the data generated, and critically interpreting results against the literature. Experience with all of these steps directly enhances critical thinking and problem-solving skills. Furthermore, the exchanges between undergraduate students, their advisors, and peers working on other aspects of the same scientific problem, and the presentation of their work at scientific conferences ultimately foster a more holistic mindset, enabling them to view the scientific problem in question within the context of the "big picture." An undergraduate research experience in the early years of school will equip students with tools for their clinical careers. By becoming proficient in research, medical students will better understand the scientific basis of diagnosis and treatment and will be trained to stay current with scientific discoveries, resulting in better patient care.^( 3 – 5 )^

The skills developed doing research are vital for medical practice, where physicians must constantly evaluate information, diagnose and make informed decisions about patients’ lifes. Research training introduces students to the principles of evidence-based medicine, teaching them to critically evaluate the scientific literature, quality and rigor of studies, and the extent to which conclusions are supported by the results presented. It also teaches them how to incorporate research findings into their clinical practice. This is crucial in an era where medical information is constantly evolving and patients are increasingly exposed to online information, often of dubious quality. Exposure to research can spark a passion for discovery and encourage students to think critically about the "why" of medical practices. This can lead to innovative ideas and a desire to contribute to the advancement of medical knowledge, but most importantly, to the understanding that evidence-based medicine is the only way to ensure patients receive the best care.^( 6 )^

Research experience can make medical students more attractive to residency programs and improve their competitiveness for fellowships and jobs, both academic and in clinical practice. It also suggests a commitment to lifelong learning and professional development, which are highly valued by employers these days. Integrating research training into medical education can foster a culture of scientific inquiry and continuous improvement in the medical field. Research experience is not just an academic pursuit; it is an essential component of medical education that prepares students to be competent, curious, and innovative clinicians, ultimately benefiting both themselves and their patients.^( 7 )^

In summary, scientific training during Medical School is not a necessity only for those physicians who intend to pursue academic careers or to pursue research in industry, but is a fundamental factor in the training of all medical students and students in all healthcare in general.
